# Mechanical ventilation during experimental sepsis increases deposition of advanced glycation end products and myocardial inflammation

**DOI:** 10.1186/cc7911

**Published:** 2009-06-09

**Authors:** Martin CJ Kneyber, Roel P Gazendam, Hans WM Niessen, Jan-Willem Kuiper, Claudia C Dos Santos, Arthur S Slutsky, Frans B Plötz

**Affiliations:** 1 Department of Pediatric Intensive Care, VU university medical center, P.O. Box 7057, 1007 MB Amsterdam, The Netherlands; 2 Institute for Cardiovascular Research (ICaR-VU), VU university medical center, P.O. Box 7057, 1007 MB, Amsterdam, The Netherlands; 3 Beatrix Children's Hospital/University Medical Center, P.O. Box 30001, 9700 RB Groningen, The Netherlands; 4 Department of Pathology, VU university medical center, P.O. Box 7057, 1007 MB Amsterdam, The Netherlands; 5 Department of Cardiac Surgery, VU university medical center, P.O. Box 7057, 1007 MB, Amsterdam, The Netherlands; 6 Keenan Research Centre, Li Ka Shing Knowledge Institute, St. Michael's Hospital, 30 Bond Street, Toronto, Ontario, M5B 1W8; University of Toronto, Toronto, Ontario, Canada

## Abstract

**Introduction:**

Increasing evidence links advanced glycation end products (AGE) including N^ε^-(carboxymethyl)lysine (CML) to the development of heart failure. Accumulation of AGE leads to myocardial inflammation, which is considered as one of the possible mechanisms underlying sepsis-induced cardiac dysfunction. We hypothesized that mechanical ventilation (MV) augmented sepsis-induced myocardial CML deposition and inflammation.

**Methods:**

Sepsis was induced using a modified cecal ligation and perforation (CLP) technique in 36 male adult Sprague Dawley rats. Rats were randomized to four hours of MV with low tidal volume (LTV: 6 ml/kg, PEEP 5 cmH_2_O, n = 10) or high tidal volume (HTV: 15 ml/kg, PEEP 3 cmH_2_O, n = 10) 24 hours after the induction of sepsis. Eight rats served as septic, non-ventilated controls and eight as non-septic, non-ventilated controls. After 28 hours all rats were killed. The number of extravascular polymorphonuclear (PMN) leucocytes, macrophages, and lymphocytes was measured as the number of positive cells/mm^2^. The number of CML positive endothelial cells were semi-quantified based upon an intensity score. The CML intensity score was correlated with the number of inflammatory cells to study the association between CML depositions and inflammation.

**Results:**

Gas exchange was comparable between the ventilated groups. Sepsis induced a significant increase in CML deposition in both ventricles that was significantly augmented by MV compared with non-ventilated septic controls (left ventricle 1.1 ± 1.0 vs 0.7 ± 0.1, *P *= 0.030; right ventricle 2.5 ± 0.5 vs 0.6 ± 0.1, *P *= 0.037), irrespective of ventilatory strategy. In the right ventricle there was a non-significant tendency towards increased CML deposition in the HTV group compared with septic, non-ventilated controls (1.0 ± 0.1 vs 0.7 ± 0.09, *P *= 0.07). Sepsis induced a significant increase in the number of macrophages and PMNs compared with non-ventilated septic controls that was augmented by MV, irrespective of ventilatory strategy. CML deposition was significantly correlated with the number of macrophages and PMNs in the heart.

**Conclusions:**

Sepsis induces CML deposition in the heart with a predominant right ventricular inflammation that is significantly augmented by MV, irrespective of the ventilatory strategy.

## Introduction

Sepsis-induced cardiac dysfunction occurs in approximately 40 to 50% of patients with prolonged septic shock and is associated with increased mortality. Various possible underlying mechanisms have been reviewed extensively [1-6]. Myocardial inflammation is one of these mechanisms as sepsis-induced cellular infiltration of the myocardium has been found in experimental studies and in humans dying from sepsis [7-9].

Advanced glycation end products (AGE) such as N^ε^-(carboxymethyl)lysine (CML) may play an important role in this inflammation [10]. AGE are formed during oxidative stress, acting as ligands for AGE receptors (RAGE) [11]. These receptors trigger a cascade of signaling mechanisms with subsequent expression of vascular cell adhesion molecule-1 (VCAM-1), induction of vascular leakage, and increased chemotaxis of mononuclear phagocytes and release of pro-inflammatory mediators resulting in cellular dysfunction [12-15]. The soluble form of the receptor (sRAGE) was found to be elevated in septic patients and associated with worsened outcome [16].

The detrimental effect of AGE may be enhanced by mechanical ventilation (MV) (double-hit principle). MV induces inflammation of healthy lungs or aggravates pre-existing lung injury (ventilator-induced lung injury); various mediators produced during this inflammation including AGE formed during MV-induced oxidative stress may contribute to distant organ failure, including the heart [17-21].

We hypothesized that sepsis led to myocardial CML deposition resulting in inflammation. In addition, we hypothesized that this effect was augmented following four hours of MV. To test this hypothesis we therefore designed a study in which rats were subjected to two different ventilatory strategies in a model of sepsis induced by cecal ligation and puncture (CLP).

## Materials and methods

### Animal preparation and experimental protocol

All animals were treated according to the Canadian national guidelines and with approval of the Institutional Animal Care and Use Committee of St Michael's Hospital. Sepsis was induced in Sprague Dawley rats (weight ± 300 g; Charles Rivers, St Constan, QC, Canada) using a modification of the cecal ligation and perforation technique [22,23]. Twenty-four hours later, rats were randomized to one of two strategies and ventilated for four hours: low tidal volume (LTV) of 6 ml/kg and positive end-expiratory pressure (PEEP) 5 cm H_2_O (n = 10); or high tidal volume (HTV) of 15 ml/kg and PEEP 3 cm H_2_O (n = 10). Normocapnia (partial pressure of arterial carbon dioxide (PaCO_2_) 35 to 45 mmHg) was maintained by adjusting respiratory rate. Inspiratory to expiration time was set to 1:2. The fraction of inspired oxygen was 0.4 in both ventilated groups. Anesthesia was maintained with intravenous xylazine 1 mg/kg/hr and ketamine 20 mg/kg/hr; muscle relaxation was achieved by continuous intravenous administration of pancuronium bromide (Sabex Inc, QC, Canada) 0.6 mg/kg/hr. For blood sampling and arterial blood pressure measurements, a catheter was inserted into the right carotid artery. All rats received a continuous infusion of normal saline at a rate of 10 ml/kg/hr to keep mean arterial pressure above 60 mmHg. At the end of the experiment animals were sacrificed with an overdose of anesthesia. Part of the lung was weighed and heated overnight to determine lung wet-to-dry ratio.

Eight rats that underwent the CLP procedure were not subjected to MV. Another eight rats were not subjected to CLP or MV. All of these animals were sacrificed after 28 hours; the first group served as non-ventilated septic controls, and the second group as non-ventilated, non-septic controls. The reported investigations were performed as part of experimental studies investigating the effects of MV during sepsis on renal function.

### Immunohistochemistry

Antibodies used were monoclonal mouse anti-rat CD68 (Serotec, Kidlington, UK), monoclonal mouse anti-rat CD45 (BD Pharmingen, Breda, The Netherlands), polyclonal rabbit anti-human myeloperoxidase (MPO) (Dako, Heverlee, Belgium) and anti-rat CML.

Hearts were fixed in 4% formaldehyde, imbedded in paraffin, and 4 μm sections were mounted on SuperFrost Plus glass slides (Menzel-Gläser, Baunschweig, Germany). The slides were deparaffinised, hydrated, and endogenous peroxidase activity was blocked by 0.03% hydrogen peroxide in methanol for 30 minutes. Enzymatic CD68 and CML antigen retrieval was performed by incubating the tissue samples with 0.1% pepsin (activated with hydrochloric acid 37%, 1:600) for 30 minutes at 37°C. MPO and CD45 heat antigen retrieval was performed by heating the slides for 15 minutes in citrate (MPO; pH 6.0) and in Tris/EDTA (CD45; pH 9.0) at 100°C. After washing the sections in demineralised water and in PBS (pH 7.4), the slides were incubated with specific antibody solutions (diluted in PBS-BSA) for 60 minutes (anti CD68 1:100, anti MPO 1:500, anti CD45 1:50, anti CML 1:500). Thereafter, the slides were again washed in PBS, followed by 30 minute incubation with anti-rabbit and anti-mouse EnVision-HRP (DakoCytomation, Heverlee, Belgium). The slides were then washed in PBS, and visualisation was performed with EnVision-diaminobenzidin (DakoCytomation, Heverlee, Belgium) for 10 minutes. Slides were counterstained with hematoxylin and mounted with Depex (Serva, Heidelberg, Germany).

As a control, the same staining procedures were used, but instead of the primary monoclonal or polyclonal antibody, PBS or an irrelevant antibody was used; these heart tissue slides were found to be negative.

### Morphometrical analyses

In each tissue slide the number of extravascular polymorphonuclear (PMN) leucocytes (MPO positive), macrophages (CD68 positive), and lymphocytes (CD45 positive) were measured as the number of positive cells/mm^2 ^myocardium using Q-PRODIT (Leica, Cambridge, UK).

The number of CML-positive endothelial cells were semi-quantified based on an intensity score for each positive vessel as follows: 1 = weak positivity; 2 = moderate positivity; 3 = strong positivity [24]. Each intensity score was multiplied by the number of vessels positive for this score. The multiplication scores were then added and the sum was divided by the area of the slide, resulting in a immunohistochemical score per mm^2^.

All morphometrical analyses were performed by two independent investigators (RPG and HWMN) who were blinded to the experimental groups. The interobserver variation was 10%.

### Statistical analysis

Data are expressed as mean ± standard error of the mean unless stated otherwise. One-way analysis of variance with Sidak *post-hoc *testing was used to analyze differences between groups. Pearson correlation coefficient was calculated to analyze correlations between continuous variables. A *P *< 0.05 was accepted as statistically significant. All statistical analyses were performed with SPSS version 17 for Macintosh (Chicago, IL, USA).

## Results

### Gas exchange and hemodynamic parameters

Gas exchange and hemodynamics were similar between the ventilated groups throughout the experiment except for a lower PaCO_2 _in the HTV group. Mean arterial blood pressure and heart rate, as well as the total amount of fluid administered during the study was no different between the LTV and HTV group. Lung wet-to-dry ratio was significantly higher in the HTV group (5.5 ± 0.1) compared with non-ventilated sepsis (4.8 ± 0.1; *P *< 0.001), but not significantly different from the non-septic group (5.0 ± 0.1).

### CML depositions

**Figure 1:**
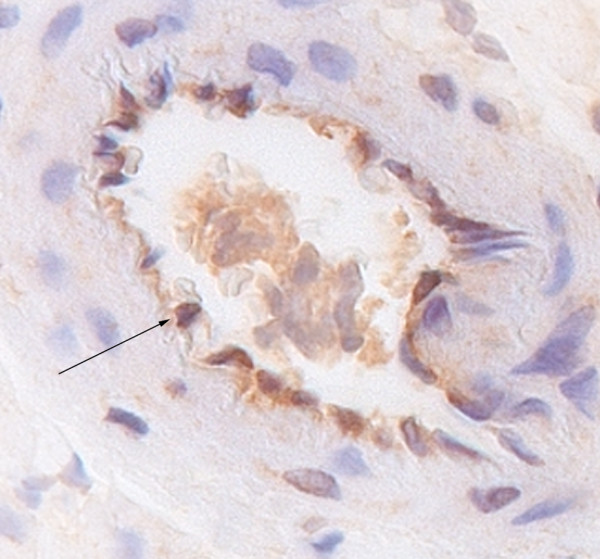
Immunohistochemical staining for N^ε^-(carboxymethyl)lysine (CML) in the left ventricular wall of a mechanically ventilated rat with sepsis. Arrows indicate positive staining of blood vessels for CML (original magnification 200×).

**Figure 2:**
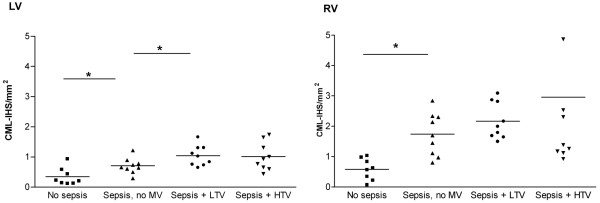
N^ε^-(carboxymethyl)lysine (CML) deposition intensity score (CML-IHS per mm^2^). Results are shown in the left ventricle (left figure) and right ventricle (right figure) of non-septic non-ventilated rats (no sepsis; n = 8), non-ventilated sepsis rats (sepsis; n = 8), rats ventilated with low tidal volume (sepsis + LTV; n = 10), and rats ventilated with high tidal volume (sepsis + HTV; n = 10). HTV = high tidal volume; LV = left ventricle; LTV = low tidal volume; MV = mechanical ventilation; RV = right ventricle. * *P *< 0.05.

CML depositions were found in small intra-myocardial arteries (Figure 1). Sepsis induced a significant increase of CML intensity score in both left ventricle (LV) and right ventricle (RV), compared with non-septic controls (Figure 2). The CML intensity score in the RV was significantly higher compared with the LV (0.7 ± 0.1 vs 1.7 ± 0.2; *P *< 0.001). The combination of sepsis and MV significantly increased the CML intensity score in both the LV and RV compared with non-septic controls (LV 0.3 ± 0.1 vs 1.0 ± 0.1, *P *< 0.01; RV 0.6 ± 0.1 vs 2.5 ± 0.5, *P *= 0.03). There were no differences between the LTV and HTV groups in both ventricles, although in the RV there was a trend towards a higher intensity score in the HTV group compared with non-ventilated sepsis (1.0 ± 0.1 vs 0.7 ± 0.09, *P *= 0.07; Figure 2).

### Myocardial inflammation

**Figure 3:**
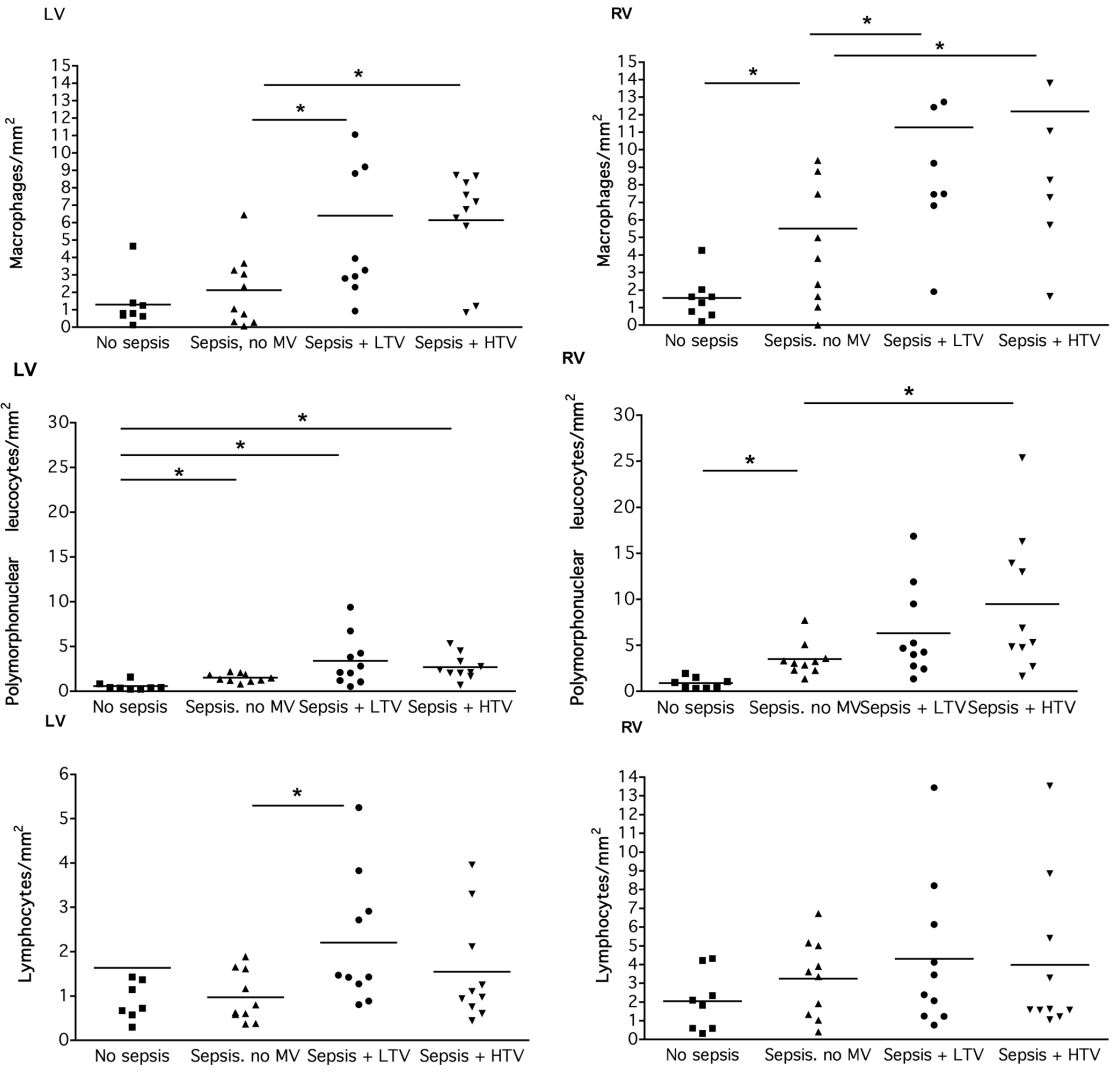
Interstitial inflammation in the myocardium. Results are shown in non-septic non-ventilated rats (no sepsis; n = 8), non-ventilated sepsis rats (sepsis; n = 8), rats ventilated with low tidal volume (sepsis + LTV; n = 10), and rats ventilated with high tidal volume (sepsis + HTV; n = 10). Upper panel depicts the number of macrophages, middle panel represents the number of polymorphonuclear leukocytes, and the lower panel represents the number of lymphocytes. HTV = high tidal volume; LV = left ventricle; LTV = low tidal volume; MV = mechanical ventilation; RV = right ventricle. * *P *< 0.05.

The CML intensity score was significantly increased by sepsis following four hours of MV, so we examined whether this was associated with increased myocardial inflammation. Sepsis did induce a significant increase of the number of macrophages in the RV (5.5 ± 1.5 vs 1.5 ± 0.4 cells/mm^2^, *P *= 0.031) but not the LV (Figure 3). MV then caused a significant increase in the number of macrophages in both the left (LTV 6.4 ± 1.8 cells/mm^2^, HTV 6.1 ± 0.9 cells/mm^2^, non-ventilated sepsis 2.1 ± 0.6 cells/mm^2^, *P *< 0.05) and RV (LTV 11.3 ± 1.9 cells/mm^2^, HTV 12.2 ± 2.1 cells/mm^2^; non-ventilated sepsis 5.5 ± 1.5 cells/mm^2^, *P *< 0.001; Figure 3). In general, the number of macrophages was significantly higher in the RV compared with the LV, except in the HTV group.

In contrast, sepsis induced a significant increase of PMNs in both left (1.5 ± 0.1 cells/mm^2 ^vs 0.6 ± 0.2 cells/mm^2^, *P *= 0.004) and RV (3.5 ± 0.6 cells/mm^2 ^vs 0.9 ± 0.2 cells/mm^2^, *P *= 0.003) compared with non-septic controls (Figure 3). However, ventilation only caused a significant increase of PMNs in the RV in the HTV group only compared with non-ventilated sepsis (9.5 ± 2.4 vs 3.5 ± 0.6 cells/mm^2^, *P *= 0.034). The number of PMNs was significantly higher in the RV compared with the LV, except in the LTV group.

The number of lymphocytes was not significantly increased in both ventricles, although for non-ventilated sepsis this was significantly higher in the RV than in the LV (Figure 3).

### Correlation of CML intensity score and myocardial inflammation

To study whether CML depositions were associated with myocardial inflammation, we studied the correlation between CML intensity score and number of inflammatory cells. CML intensity score was significantly correlated with the number of macrophages in the LV (R^2 ^= 0.14, *P *= 0.029) and number of PMNs in both ventricles (LV R^2 ^= 0.11, *P *= 0.049 and RV R^2 ^= 0.67, *P *< 0.001). There was no correlation with the number of lymphocytes.

## Discussion

The major finding of this study is that MV in combination with sepsis was associated with increased CML depositions in small intra-myocardial arteries and myocardial inflammation by macrophages and PMNs with a predominance in the RV, irrespective of ventilatory strategy.

AGE and their receptor RAGE have been identified as a pathophysiologic contributors to cellular inflammation in sepsis by amplifying the host innate immune response [10]. Our findings on increased CML depositions in both LV and RV, as well as a significant correlation between CML deposition and the number of macrophages and PMNs is in line with these observations. The low number of lymphocytes in the heart of our study may be explained by the observation of increased apoptosis of lymphocytes during sepsis [25]. Weber and colleagues have found that this apoptosis already occurs during the early phase of sepsis [26].

Our group is the first to study the pathophysiologic role of AGE formation in MV-induced myocardial inflammation during sepsis. Importantly, the role of AGE in increasing inflammation and subsequent detrimental effects on cardiovascular function has recently been firmly underscored [27]. Increased CML depositions were found in the LV and RV of ventilated animals, although ventilatory strategy itself did not have a significant effect on CML deposition as well as myocardial inflammation. This may be explained by the fact that our model only caused mild lung injury. Although the wet-to-dry ratio was significantly different between LTV and HTV, gas exchange (including partial pressure of arterial oxygen (PaO_2_) and PaCO_2_) was comparable and the wet-to-dry ratio in the HTV group was only 5.5 [28].

We can only speculate on a possible causal link between CML deposition, myocardial inflammation and MV. Non-injurious MV is associated with oxidative stress that is characterised by pulmonary production of AGE and increased oxidant released as measured by serum isoprostane [18,20,21]. Increased levels of the RAGE has been found in broncho-alveolar lavage fluids and serum of rats with endotoxin-induced lung injury and patients with acute lung injury [29]. Treatment with sRAGE significantly attenuated the increase in neutrophil infiltration, lung permeability, production of inflammatory cytokines, nuclear factor (NF)-κB activation, and apoptotic cells in the lungs [21]. The question is how local pulmonary inflammation with oxidative stress leads to accumulation of CML in distant organs. AGE such as CML can be formed by different processes including glycation followed by oxidative cleavage of Amadori-adducts, auto-oxidative glycosylation, reaction of proteins with non-glucose carbohydrates, lipoxidation, and by reaction of proteins with products of MPO derived from neutrophils [30-33]. MPO rapidly uses hydrogen peroxide to form hypochlorous acid which reacts with the amino group of free amino acids to form glycoaldehydes [30]. It may be hypothesized that the accumulation of AGE in our study may at least in part be mediated by neutrophils as these inflammatory cells play a dominant role in both sepsis and ventilator-induced lung injury [34,35]. Importantly, to ascertain a causative role for AGE on myocardial inflammation future studies should be performed investigating the effect of sRAGE or AGE formation blockers.

Although we did not study the functional correlation of sepsis-induced CML depositions and myocardial inflammation augmented by MV, it seems logical to hypothesize that this could contribute to sepsis-induced myocardial dysfunction. Accumulation of AGE are increasingly linked to the development of heart failure [36]. RAGE induces VCAM-1, used by PMNs to adhere to and infiltrate the myocardium during endotoxemia with subsequent oxidative and nitrosative stress leading to myocardial dysfunction in an experimental study [37]. In addition, Granton and colleagues studied myocardial contractility perfusing hearts from endotoxemic animals in a Langendorff set-up [38]. They found improved myocardial contractility in hearts perfused with leukocyte-depleted blood animals compared with untreated blood. Therefore, our findings require follow-up by an *in vivo *study of the effects of increased interstitial inflammation on myocardial contractility.

Interestingly, mainly the RV was involved. For instance, the correlation between RV CML depositions and number of PMNs was highly significant. This may be explained by the fact that during sepsis, and during MV of non-injured lungs the pulmonary vascular resistance is increased that may lead to increased strain with inflammation on the RV with subsequent right ventricular dysfunction [39]. This assumption is supported by both animal and human studies on pulmonary embolism with pulmonary hypertension, in which a massive accumulation of neutrophils and monocytes/macrophages has been observed [40,41]. Begieneman and colleagues found increased inflammation by macrophages and PMNs in the RV of patients dying from pulmonary embolism [40]. On the other hand, the low level of PEEP in the HTV group causing atelectasis may have confounded our results as this has been demonstrated to be associated with right ventricular failure in rats [42].

There are some methodological aspects of our study that deserve comment. For our sepsis model we used the CLP technique, which is comparable to sepsis in humans [43]. Because patients with sepsis often need to be mechanically ventilated, we constructed a double-hit model combining CLP-induced sepsis followed by MV. Tidal volumes were chosen based on clinical practice: a tidal volume of 6 ml/kg is recommended as lung-protective ventilation, and HTV is still used for MV in patients [44,45]. Mean airway pressure and PaO_2 _were similar between the LTV and HTV group, eliminating oxygenation as a possible confounding factor between these two groups. Lastly, theoretically the anesthetics used in this study could interfere with myocardial function [46]. Nevertheless, in our study hemodynamic parameters were comparable between all groups.

## Conclusions

MV during experimental sepsis was associated with increased CML depositions in intra-myocardial small arteries and myocardial inflammation, irrespective of ventilatory strategy.

## Key messages

• CML depositions in the heart were significantly increased during experimental sepsis.

• MV in combination with sepsis was associated with increased CML depositions in intra-myocardial small arteries and myocardial inflammation, irrespective of ventilatory strategy.

• The RV was predominantly affected.

## Abbreviations

AGE: advanced glycation end product; BSA: bovine serum albumin; CLP: cecal ligation and perforation; CML: N^ε^-(carboxymethyl)lysine; HTV: high tidal volume; LTV: low tidal volume; LV: light ventricle; MPO: myeloperoxidase; MV: mechanical ventilation; PaCO_2_: partial pressure of arterial carbon dioxide; PaO_2_: partial pressure of arterial oxygen; PBS: phosphate-buffered saline; PEEP: positive end-expiratory pressure; PMN: polymorphonuclear leucocytes; RAGE: receptor for advanced glycation end product; RV: right ventricle; sRAGE: soluble receptor for advanced glycation end product; VCAM-1: vascular cell adhesion molecule-1.

## Competing interests

The authors declare that they have no competing interests.

## Authors' contributions

MK designed the study, performed the statistical analysis, and wrote the first draft of the paper. RG performed the immunohistochemistry and morphometrical analysis, and performed the statistical analysis. HN supervised the immunohistochemistry and morphometrical analysis and contributed to the writing of the paper. JWK performed the animal experiments and contributed to the writing of the paper. CDS supervised the animal experiments and contributed to the writing of the paper. AS supervised the animal experiments and contributed to the writing of the paper. FP co-designed the study and contributed to the writing of the paper.
